# Accuracy of intraoral scans of the edentulous maxilla – an in vitro study

**DOI:** 10.1007/s00784-025-06419-w

**Published:** 2025-06-17

**Authors:** Panagiotis Kontis, Jan-Frederik Güth, Christine Keul

**Affiliations:** 1 Department of Prosthetic Dentistry, University Hospital, LMU Munich, Goethestrasse 70, Munich, 80336 Germany; 2 Department of Prosthetic Dentistry, Center for Dentistry and Oral Medicine (Carolinum), Goethe-University Frankfurt Am Main, Frankfurt Am Main, D-60596 Germany

**Keywords:** Accuracy, Coordinate-based data analysis, Digital dentistry, Intraoral scanner, Precision, Trueness, Digital full-arch impression, Edentulous

## Abstract

**Objective:**

Investigation of the accuracy of various digitalization methods and the accuracy of digitalization of different regions of the edentulous maxilla.

**Material and method:**

A PEEK edentulous maxilla with four spherical reference geometries served as the testing model. A reference dataset (REF) was generated using a highly accurate 3D measuring instrument. The testing model was digitized as follows (*n* = 25/group). Direct digitalization (DD) with intraoral scanners (IOS): 1) Cerec AC Primescan (PRI), 2) Trios 4 Move (TR4), 3) Trios 3 Wireless (TR3), 4) Indirect digitalization of PVS impression with laboratory scanner 3Shape D810 (D8I). Three-dimensional deviations between REF and TEST were evaluated (GOM Inspect) in different areas of the model: 1) Complete Surface, 2) Alveolar Ridge, 3) Vestibular Ridge, 4) Palate, 5) Posterior Seal, 6) Border. Significant differences were analyzed with the Games-Howell test for trueness (*p* < 0.05) and multiple comparisons Levene's test for precision (for IOS: *p* < 0.008, for area: *p* < 0.003).

**Results:**

Group D8I revealed the best trueness for Complete Surface (7.95 µm), Palate (9.11 µm), and Border (20.22 µm). Alveolar Ridge showed for PRI (16.45 µm) and TR4 (8.96 µm) the highest trueness. Groups TR4 and PRI resulted in significantly higher precision for Alveolar Ridge. Groups TR4 and D8I demonstrated the highest precision for Palate. Complete Surface and Alveolar Ridge showed for all digitalization methods significantly higher precision.

**Conclusions:**

Indirect digitalization of impressions remains the most accurate approach for capturing edentulous jaws, whereas IOS deliver datasets with clinically acceptable accuracy. Peripheral regions characterized by limited accessibility and smooth surface morphology tend to demonstrate increased deviations in the resulting digital datasets.

**Clinical relevance.:**

Indirect digitalization of the impression still appears to be the most appropriate technique to access the clinical workflow for full dentures due to the superior digitalization trueness and inclusion of functional movements. Direct and indirect digitalization show nearly equal values for precision.

## Introduction

Over the past decades technological innovations in dental workflows and materials have profoundly transformed diverse aspects of the profession. Apart from the field of fixed prosthodontics, CAD/CAM processes have numerous applications in one of the oldest therapeutic practices in dentistry, namely the complete denture [1, 2]. Computer assisted design with the use of dedicated software, digital articulators, expansive teeth libraries, and seamless combination of face scans, patient’s photographs and digital models offer improved efficiency, reduced time, and cost [3, 4]. Simultaneously, novel polymer materials used to mill the basis or teeth of the denture provide higher stability, better biocompatibility, less microorganism adhesion and result in better base adaptation, lower incidence of pressure points and correction appointments [3–5].

Intraoral scanners (IOS) have been well established for the digitalization of fully dentate jaws and have been proven to be as accurate as conventional impressions for the entire arch in certain clinical scenarios [6, 7]. Still CAD/CAM workflows for complete dentures rely mainly on conventional impressions or casts for the acquisition of the edentulous anatomy [2]. When applying IOS for completely edentulous situations the main limitations described are the scarcity of anatomical landmarks, the functional borders, and the posterior palatal seal [2, 8]. In fully dentate arches the tooth morphology is utilized by the superimposition algorithm to correctly align the captured images. For edentulous areas this process cannot be carried out as efficiently due to the lack of dental geometries. This in turn can result in larger inaccuracies and deformities in the digital model [9–11]. Furthermore, IOS achieve a purely mucostatic record of the intraoral anatomy, whereas indirect digitalization includes information about functional muscle extension, vibrating line spread and depth or displacement of mucosal areas of varying compressibility [12–14]. These features have long been regarded as key components for the realization of adequate retention and stability in complete prostheses [15, 16]. Recently many researchers have theorized that for CAD/CAM milled dentures, despite IOS data lacking peripheral seal information, the accuracy of digitalization may be sufficient to achieve high conformity between the denture base and the underlying mucosa, ensuring adequate retention according to the mucostatic principle [13, 17–20].

However, the current body of evidence on the accuracy of direct digitalization of edentulous jaws remains limited, both in terms of volume and content of available research [8, 21]. The determination of the accuracy of digital datasets for edentulous jaws presents some methodological complexities and requires a three-dimensional comparison of the digital datasets with the anatomy of the denture-bearing area. Previous investigations have mostly focused on comparisons of IOS and digitized conventional impressions, without the use of highly accurate reference datasets [12, 13, 22–25]. Furthermore, researchers almost exclusively employ global best fit algorithms for the orientation of data, which has been proven to obscure true inaccuracies and result in potentially lower deviations, especially regarding larger datasets [26]. Moreover, comparisons of the accuracy between various areas of the edentulous digital models are usually relayed with color coded maps of selected representative datasets, without quantitative analysis on the entirety of generated data or statistical evaluation of the results [12, 23, 27–29]. These differences in the methodological design complicate the comparison of the study results with each other.

The aim of the present in vitro study is to assess the accuracy (trueness and precision) of direct and indirect digitalization on an edentulous maxillary model as well as the accuracy of digitalization for different parts of the edentulous maxilla. The first null hypothesis is that there are no significant differences in the accuracy of different digitalization methods. The second null hypothesis states that there are no significant differences in the accuracy of varying areas of the digital edentulous maxillary models achieved with each method.

## Materials and methods

### Testing model

**Fig. 1:**
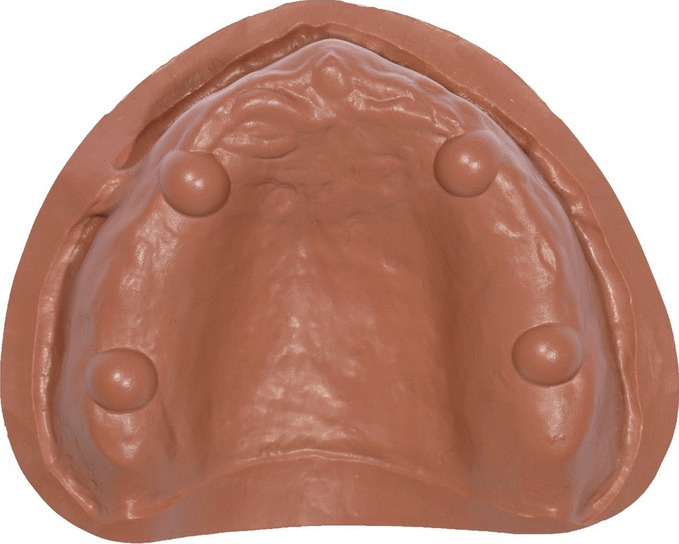
Testing model milled from PEEK

**Fig. 2:**
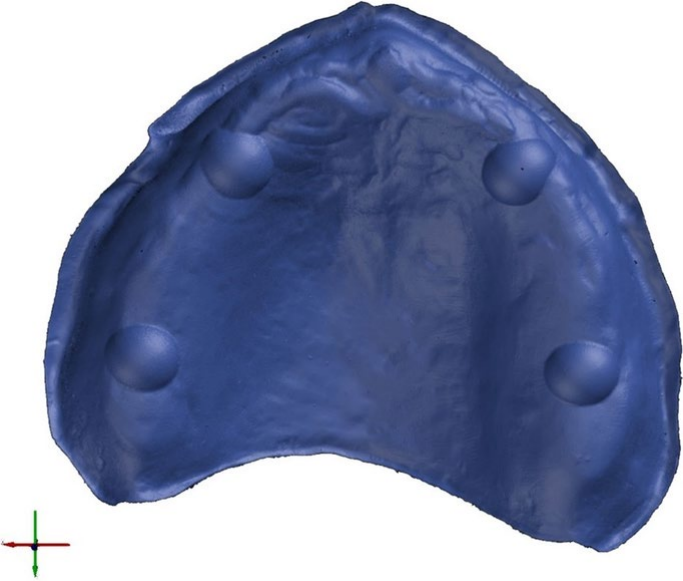
Reference dataset

An edentulous maxillary model with four identical hemispheres in the location of the canines and second molars on the residual ridge was milled from PEEK (Polyether ether ketone) (PEEK Biosolution, LOT no 32116; Merz Dental GmbH, Lütjenberg, Germany) and used as the testing model (Fig. 1). To produce a highly accurate reference dataset (REF, Fig. 2), the testing model was optically measured using the Infinite Focus G5 scanner (Alicona Imaging GmbH, Graz, Austria; objective 5x, resolution: finest topographic lateral = 3.51 µm, vertical = 410 nm).

### Direct digitalization

Direct digitalization of the PEEK testing model was completed with the following IOS (n = 25/group):

- Cerec Primescan AC (group PRI; Software version 5.0.2, Dentsply Sirona, Bensheim, Germany).
- Trios 4 Move (group TR4; Software Version 1.19.2.4, 3 Shape, Copenhagen, Denmark).
- Trios 3 Wireless (group TR3; Software version 1.4.7.4, 3 Shape, Copenhagen, Denmark).

Cerec Primescan AC and Trios 3 were calibrated at the beginning of every scanning session, whereas for Trios 4 no calibration was required as per manufacturer’s recommendation. All scans were conducted by one single experienced user, with a five-minute intermission between each scan.

The following strategy was applied: first the vestibular side of the residual ridge was scanned commencing with the tuberosity of the first quadrant and concluding in the tuberosity of the second quadrant, followed by the palatal surface of the ridge in the reverse direction. Lastly the palate was recorded in a zig zag path. Test datasets were post-processed and exported as STL.

### Indirect digitalization

**Fig. 3:**
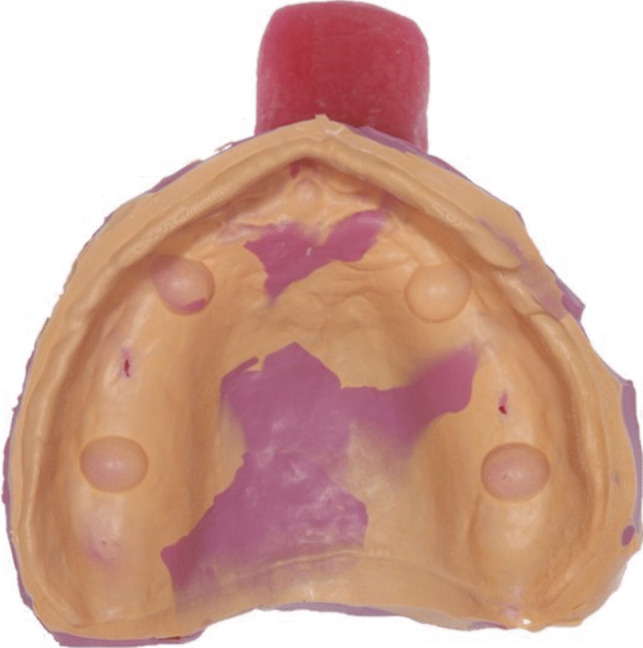
PVS impression

Twenty-five conventional impressions were conducted with scannable polyvinyl siloxane (PVS) impression material. The impressions were carried though in a one-step/double mix impression technique. Light body material (Flexitime Fast&Scan light flow, LOT no K010022; Kulzer GmbH, Hanau, Germany) was directly injected on the testing model, while medium body material (Flexitime Monophase Pro Scan, LOT no R010022; Kulzer GmbH, Hanau, Germany) was applied in the custom tray (Fig. 3). The tray was then positioned and held without pressure on the model for four minutes. After removal the impressions were disinfected (ORBI-Sept Abformdesinfektion, LOT no A0984; Orbis Dental, Münster, Germany) for two minutes, cleaned under running water and air-dried.

Following a twenty-four-hour storing period, the PVS impressions were digitized with the D810 laboratory scanner (group D8I; Software Version Dental System 2014–1 × 64—build 1.4.7.4–16.08.2018 Dental System, 3 Shape; *n* = 25/group).

### Analysis of datasets

**Fig. 4:**
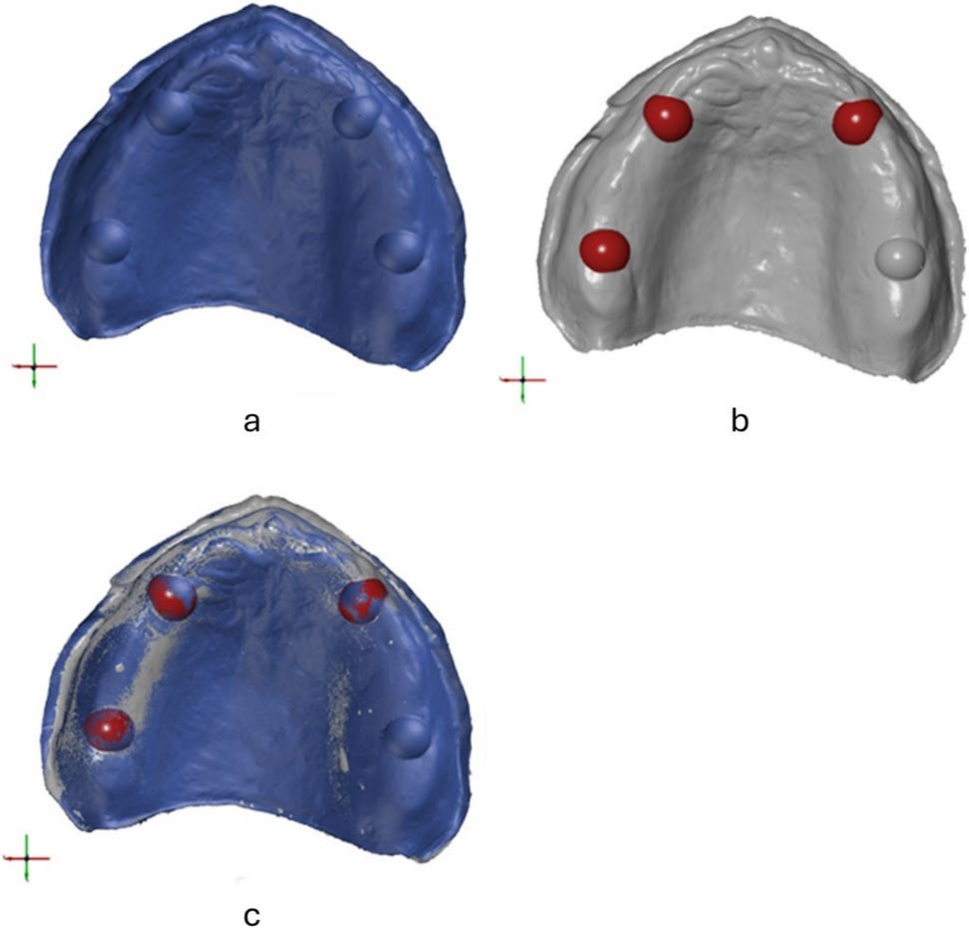
Alignment of test datasets with REF. **a**. REF. **b**. Best fit alignment on selected areas. **c**. Aligned test and REF datasets

All generated datasets as well as the reference dataset were imported into the metrological software GOM Inspect (GOM Inspect 2020, GOM GmbH, Braunschweig, Germany; software version: 133088). Artifacts were removed and margins were trimmed up to the edge of the border. For each group the test and REF datasets were aligned using a local best fit on the hemispheres located in both canine positions and the second molar of the first quadrant (Fig. 4).

**Fig. 5:**
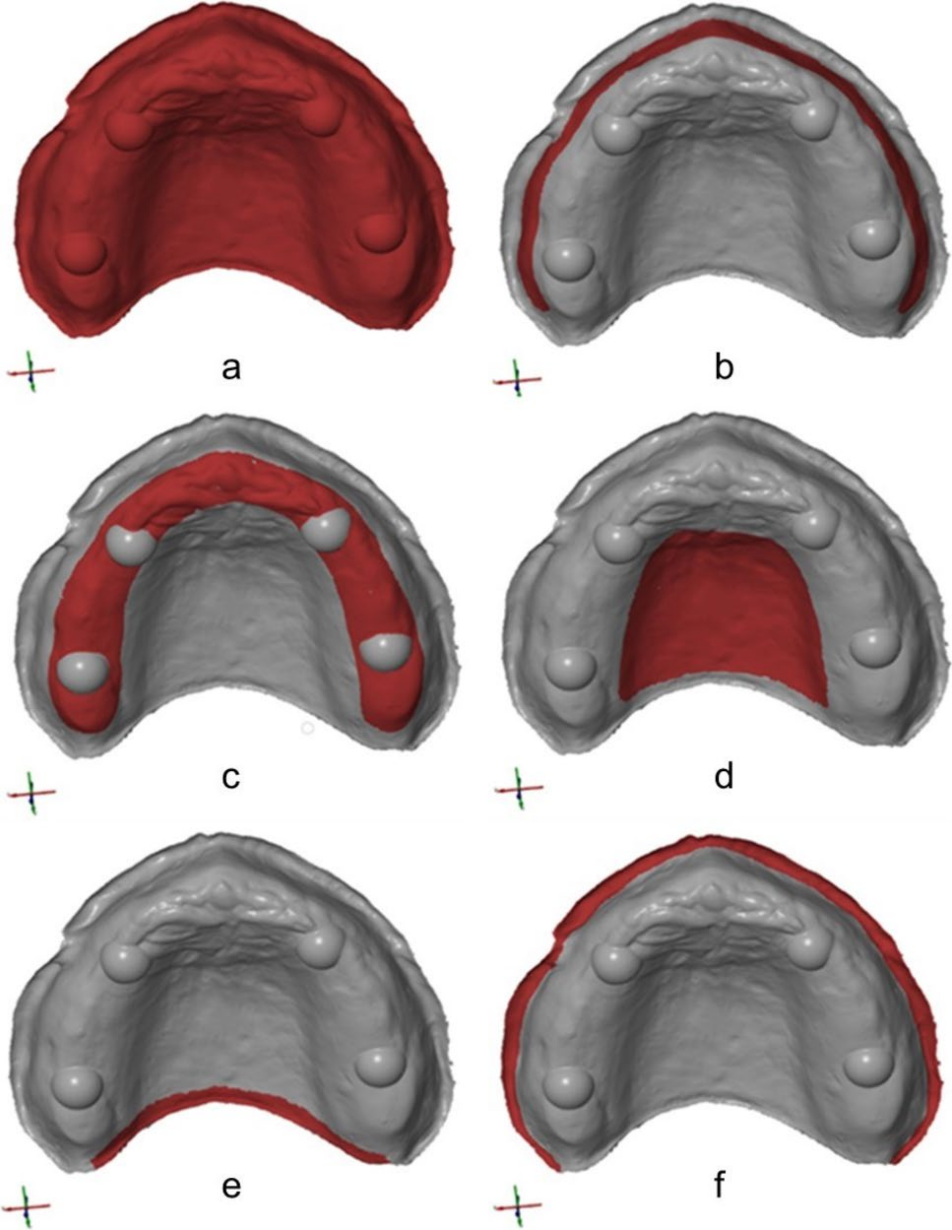
Investigated areas: **a**. Complete Surface. **b**. Vestibular Ridge. **c**. Alveolar Ridge. **d**. Palate. **e**. Posterior Seal. **f**. Border

**Fig. 6:**
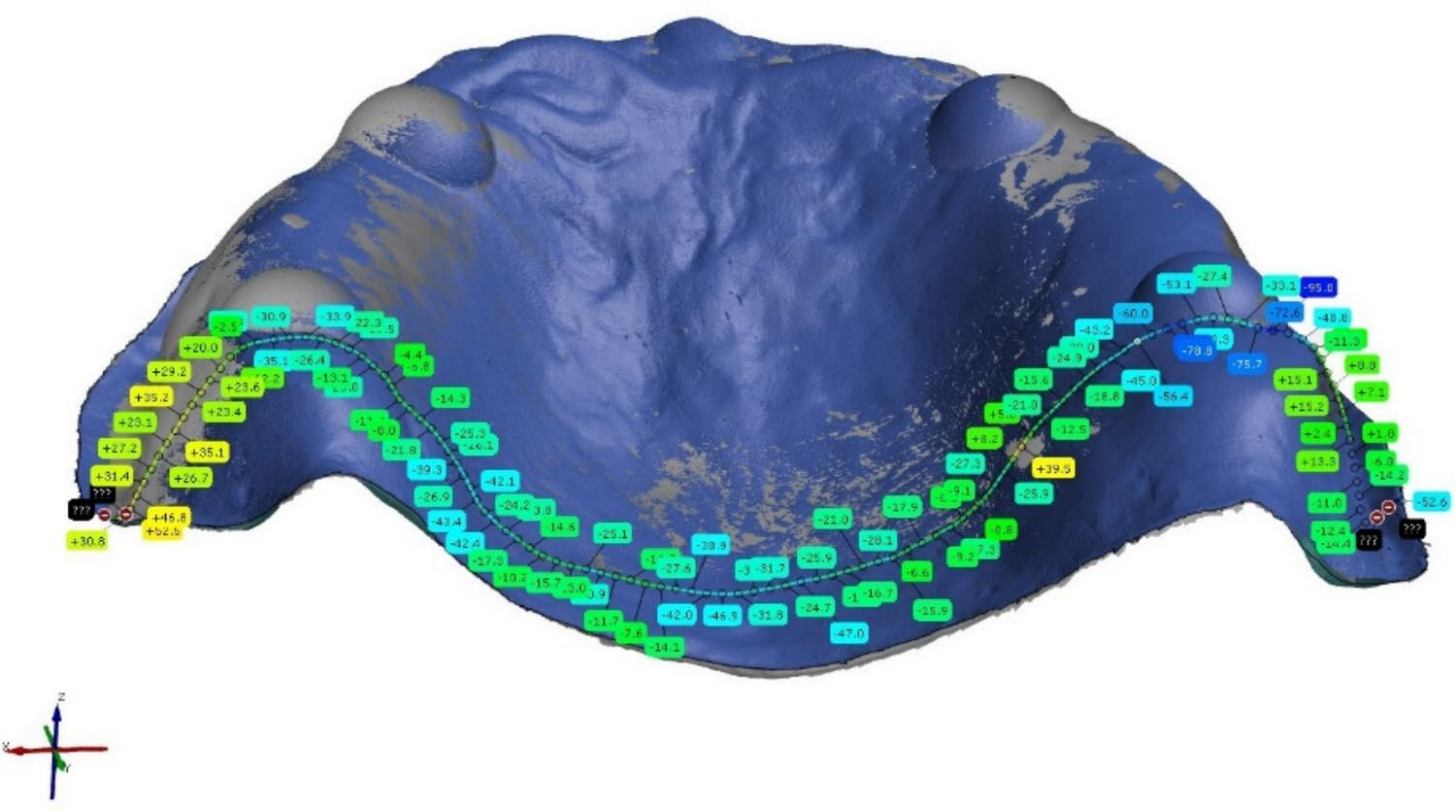
Cross section of measured distances between points on REF and test datasets in the metrological software

After alignment, the following areas were manually selected on the REF: *Complete Surface, Alveolar Ridge, Vestibular Ridge, Palate, Posterior Seal, Border* (Fig. 5). For each dataset the distances of individual points between REF and test dataset in every selected area were calculated (Fig. 6). All measured values were exported into Microsoft Excel and the median of the absolute values was determined. Accuracy of digitalization was assessed as the trueness and precision of the data as defined by the ISO 5725–1 [30]. Trueness was calculated from the difference between measured and reference data. For the examination of precision, the spread of the values represented by the standard deviation was examined.

### Statistical analysis

For statistical analysis the Statistical Package for the Social Sciences, Version 25 (SPSS Inc., Chicago, USA) was used. Descriptive statistics (mean, standard deviation, 95% confidence intervals) were given for each parameter and group. An analysis of variance (ANOVA) test was applied to assess the null hypothesis. Normality of data distribution was tested using Kolmogorov–Smirnov and Shapiro–Wilk test. Significant differences in trueness were examined by a post-hoc Games-Howell test with significance level of *p* = 0.05. Regarding precision significant differences in variance were analyzed by multiple comparison Levene’s Test, where the significance level was set at *p* = 0.008 for the digitalization method and *p* = 0.003 for the area.

## Results

**Fig. 7:**
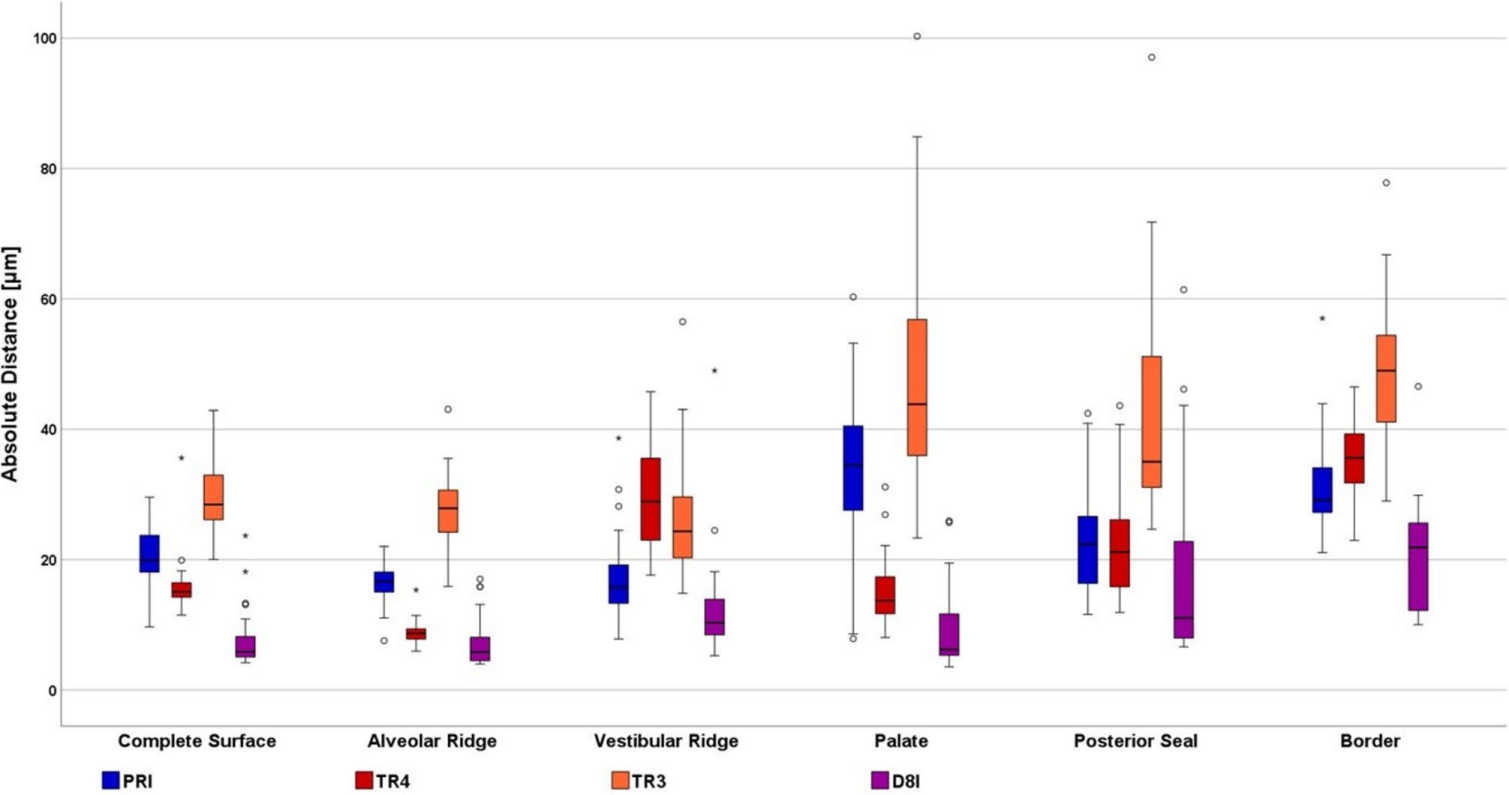
Boxplots for digitalization methods (Cerec AC Primescan (PRI), Trios 4 Move (TR4), Trios 3 Wireless (TR3), Indirect digitalization of PVS impression with laboratory scanner 3Shape D810 (D8I)) and digitalization areas (Complete Surface, Alveolar Ridge, Vestibular Ridge, Palate, Posterior Seal, Border)

Table 1 reports the descriptive statistics (mean, standard deviation (SD), median (Med), minimum (Min), maximum (Max), and 95% confidence interval (CI)) for each parameter (Tables 2 and 3). Table 2 and Table 4 depict the *p* values of the Games-Howell post-hoc test for trueness. Table 3 and Table 5 depict the *p* values of Levene’s test for precision. Figure 7 shows the boxplots.

**Table 1:** Descriptive statistics: Mean, standard deviation (SD), median (Med), minimum (Min), maximum (Max), and 95% confidence interval (CI)

Digitalization Method	Area	Absolute Distance (μm)					
		Mean	SD	Med	Min	Max	95% CI
PRI	*Complete Surface*	20.44^C, b,1, α^	4.80	19.90	9.66	29.59	18.46
	*Alveolar Ridge*	16.42^B, a, 1, 2, α^	3.25	16.64	7.56	22.03	15.08
	*Vestibular Ridge*	17.02^A, a, b, 1, α, β^	7.45	15.74	7.82	38.65	13.94
	*Palate*	32.73^C, c, 2, β^	13.85	34.47	7.86	60.28	27.02
	*Posterior Seal*	22.58^A, b, 1, β^	7.85	22.36	11.59	42.44	19.34
	*Border*	31.27^B, c, 1, α, β^	7.51	29.13	21.07	56.99	28.17
TR4	*Complete Surface*	16.04^B, b, 1, α, β^	4.45	15.07	11.49	35.61	14.20
	*Alveolar Ridge*	8.96^A, a, 1, α^	1.80	8.66	5.96	15.34	8.22
	*Vestibular Ridge*	29.13^B, c, 1, γ^	7.43	28.92	17.63	45.72	26.06
	*Palate*	15.40^B, b, 1, β, γ^	5.53	13.70	8.03	31.16	13.11
	*Posterior Seal*	23.49^A, c, 1, 2, γ^	9.61	21.17	11.90	43.58	19.53
	*Border*	35.50^B, d, 1, β, γ^	6.20	35.61	22.94	46.47	32.94
TR3	*Complete Surface*	29.94^D, a, 1, α^	5.82	28.47	20.03	42.90	27.54
	*Alveolar Ridge*	27.67^C, a, 2, α^	15.88	27.87	15.88	43.05	25.30
	*Vestibular Ridge*	27.02^B, a, 1, α, β^	9.60	24.35	14.83	56.47	23.06
	*Palate*	48.32^D, b, 2, β^	19.40	43.81	23.31	100.3	40.31
	*Posterior Seal*	42.36^B, b, 2, β^	16.97	35.03	24.67	97.07	35.35
	*Border*	48.97^C, b, 1, α, β^	11.39	48.95	29.01	77.80	44.26
D8I	*Complete Surface*	7.95^A, a, 1, α^	4.75	5.84	4.18	23.67	5.99
	*Alveolar Ridge*	7.61^A, a, 2, α^	4.08	5.80	3.98	17.01	5.93
	*Vestibular Ridge*	12.68^A, a, b, 1, α, β, γ^	8.79	10.32	5.26	48.96	9.05
	*Palate*	9.11^A, a, b, 1, α, β^	6.23	6.20	3.53	25.95	6.54
	*Posterior Seal*	18.88^A, b, c, 2, γ^	15.20	11.04	6.62	61.39	12.60
	*Border*	20.22^A, c, 1, β, γ^	8.70	21.87	10.02	46.54	16.63

Superscript upper-case letters indicate significant differences in trueness between different digitalization methods

Superscript lower-case letters indicate significant differences in trueness between areas

Superscript numbers indicate significant differences in precision between different digitalization methods

Superscript Greek lowercase letters indicate significant differences in precision between different areas

**Table 2:** *P*-values for trueness of digitalization method

	*Complete Surface*					*Palate*			
	PRI	TR4	TR3	D8I		PRI	TR4	TR3	D8I
PRI		0.008	< 0.001	< 0.001	PRI		< 0.001	0.011	< 0.001
TR4	0.008		< 0.001	< 0.001	TR4	< 0.001		< 0.001	0.002
TR3	< 0.001	< 0.001		< 0.001	TR3	0.011	< 0.001		< 0.001
D8I	< 0.001	< 0.001	< 0.001		D8I	< 0.001	0.002	< 0.001	
	*Alveolar Ridge*					*Posterior Seal*			
	PRI	TR4	TR3	D8I		PRI	TR4	TR3	D8I
PRI		< 0.001	< 0.001	< 0.001	PRI		0.983	< 0.001	0.702
TR4	< 0.001		< 0.001	0.440	TR4	0.983		< 0.001	0.578
TR3	< 0.001	< 0.001		< 0.001	TR3	< 0.001	< 0.001		< 0.001
D8I	< 0.001	0.440	< 0.001		D8I	0.702	0.578	< 0.001	
	*Vestibular Ridge*					*Border*			
	PRI	TR4	TR3	D8I		PRI	TR4	TR3	D8I
PRI		< 0.001	< 0.001	0.249	PRI		0.147	< 0.001	< 0.001
TR4	< 0.001		0.821	< 0.001	TR4	0.147		< 0.001	< 0.001
TR3	< 0.001	0.821		< 0.001	TR3	< 0.001	< 0.001		< 0.001
D8I	0.249	< 0.001	< 0.001		D8I	< 0.001	< 0.001	< 0.001

**Table 3:** *P*-values for the precision of digitalization method

	*Complete Surface*					*Palate*			
	PRI	TR4	TR3	D8I		PRI	TR4	TR3	D8I
PRI		0.143	0.303	0.644	PRI		0.001	0.237	0.003
TR4	0.143		0.025	0.319	TR4	0.001		< 0.001	0.606
TR3	0.303	0.025		0.163	TR3	0.237	< 0.001		< 0.001
D8I	0.644	0.319	0.163		D8I	0.003	0.606	< 0.001	
	*Alveolar Ridge*					*Posterior Seal*			
	PRI	TR4	TR3	D8I		PRI	TR4	TR3	D8I
PRI		0.023	0.021	0.261	PRI		0.229	0.004	0.007
TR4	0.023		< 0.001	0.001	TR4	0.229		0.033	0.060
TR3	0.021	< 0.001		0.089	TR3	0.004	0.033		0.692
D8I	0.261	0.001	0.089		D8I	0.007	0.060	0.692	
	*Vestibular Ridge*					*Border*			
	PRI	TR4	TR3	D8I		PRI	TR4	TR3	D8I
PRI		0.507	0.294	0.896	PRI		0.823	0.032	0.179
TR4	0.507		0.558	0.508	TR4	0.823		0.010	0.069
TR3	0.294	0.558		0.312	TR3	0.032	0.010		0.267
D8I	0.896	0.508	0.312		D8I	0.179	0.069	0.267

**Table 4:** *P*-values for trueness of digitalized area

	PRI					
	*Complete Surface*	*Alveolar Ridge*	*Vestibular Ridge*	*Palate*	*Posterior Seal*	*Border*
*Complete Surface*		0.014	0.397	0.003	0.852	< 0.001
*Alveolar Ridge*	0.014		0.999	< 0.001	0.012	< 0.001
*Vestibular Ridge*	0.397	0.999		< 0.001	0.124	< 0.001
*Palate*	0.003	< 0.001	< 0.001		0.032	0.997
*Posterior Seal*	0.852	0.012	0.124	0.032		0.003
*Border*	< 0.001	< 0.001	< 0.001	0.997	0.003	
	TR4					
	*Complete Surface*	*Alveolar Ridge*	*Vestibular Ridge*	*Palate*	*Posterior Seal*	*Border*
*Complete Surface*		< 0.001	< 0.001	0.977	0.015	< 0.001
*Alveolar Ridge*	< 0.001		< 0.001	< 0.001	< 0.001	< 0.001
*Vestibular Ridge*	< 0.001	< 0.001		< 0.001	0.207	0.022
*Palate*	0.997	< 0.001	< 0.001		0.021	< 0.001
*Posterior Seal*	0.015	< 0.001	0.207	< 0.001		< 0.001
*Border*	< 0.001	< 0.001	0.022	0.009	< 0.001	
	TR3					
	*Complete Surface*	*Alveolar Ridge*	*Vestibular Ridge*	*Palate*	*Posterior Seal*	*Border*
*Complete Surface*		0.735	0.782	0.001	0.019	< 0.001
*Alveolar Ridge*	0.735		1.000	< 0.001	0.004	< 0.001
*Vestibular Ridge*	0.782	1.000		< 0.001	0.004	< 0.001
*Palate*	0.001	< 0.001	< 0.001		0.855	1.000
*Posterior Seal*	0.019	0.004	0.004	0.855		0.592
*Border*	< 0.001	< 0.001	< 0.001	1.000	0.592	
	D8I					
	*Complete Surface*	*Palate*	*Vestibular Ridge*	*Palate*	*Posterior Seal*	*Border*
*Complete Surface*		1.000	0.194	0.975	0.021	< 0.001
*Alveolar Ridge*	1.000		0.121	0.913	0.015	< 0.001
*Vestibular Ridge*	0.194	0.121		0.567	0.499	0.041
*Alveolar Ridge*	0.975	0.913	0.567		0.057	< 0.001
*Posterior Seal*	0.021	0.015	0.499	0.057		0.999
*Border*	< 0.001	< 0.001	0.041	< 0.001	0.999

**Table 5:** *P*-values for precision of digitalized area

	PRI						
		*Complete Surface*	*Alveolar Ridge*	*Vestibular Ridge*	*Palate*	*Posterior Seal*	*Border*
*Complete Surface*			0.065	0.147	< 0.001	0.070	0.347
*Alveolar Ridge*		0.065		0.007	< 0.001	0.002	0.021
*Vestibular Ridge*		0.147	0.007		0.013	0.745	0.841
*Palate*		< 0.001	< 0.001	0.013		0.024	0.010
*Posterior Seal*		0.070	0.002	0.745	0.024		0.610
*Border*		0.347	0.021	0.841	0.010	0.610	
		TR4					
		*Complete Surface*	*Alveolar Ridge*	*Vestibular Ridge*	*Palate*	*Posterior Seal*	*Border*
*Complete Surface*			0.165	< 0.001	0.104	< 0.001	0.021
*Alveolar Ridge*		0.165		< 0.001	< 0.001	< 0.001	< 0.001
*Vestibular Ridge*		< 0.001	< 0.001		0.014	0.128	0.185
*Palate*		0.104	* < 0.001*	0.014		0.008	0.456
*Posterior Seal*		* < 0.001*	* < 0.001*	0.128	0.008		0.036
*Border*		0.021	* < 0.001*	0.185	0.456	0.036	
		TR3					
		Complete Surface	Alveolar Ridge	Vestibular Ridge	Palate	Posterior Seal	Border
*Complete Surface*			0.774	0.089	* < 0.001*	* < 0.001*	0.006
*Alveolar Ridge*		0.774		0.063	< 0.001	* < 0.001*	0.004
*Vestibular Ridge*		0.089	0.063		0.005	0.022	0.324
*Palate*		*< 0.001*	*< 0.001*	0.005		0.528	0.033
*Posterior Seal*		*< 0.001*	*< 0.001*	0.022	0.528		0.120
*Border*		0.006	0.004	0.324	0.033	0.120	
		D8I					
		Complete Surface	Alveolar Ridge	Vestibular Ridge	Palate	Posterior Seal	Border
*Complete Surface*			0.809	0.231	0.220	*< 0.001*	*0.002*
*Alveolar Ridge*		0.809		0.169	0.123	< 0.001	* < 0.001*
*Vestibular Ridge*		0.231	0.169		0.721	0.006	0.270
*Palate*		0.220	0.123	0.721		*< 0.001*	0.054
*Posterior Seal*		*< 0.001*	*< 0.001*	0.006	< 0.001		0.027
*Border*		*0.002*	< 0.001	0.270	0.054	0.027	

### Accuracy of digitalization method:

For *Complete Surface, Palate* and *Border* D8I exhibited the significant highest trueness while TR3 resulted in significantly lowest trueness. For *Alveolar Ridge* D8I and TR4 exhibited the highest trueness. For *Vestibular Ridge* D8I and PRI demonstrated significantly the highest trueness. For *Posterior Seal* D8I, PRI, and TR4 demonstrated significantly higher trueness than group TR3.

For *Alveolar Ridge* TR4 showed significantly higher precision than D8I and TR3. For *Palate* TR4 and D8I revealed significantly higher precision than PRI and TR3. For *Posterior Seal* PRI demonstrated significantly the highest precision. For *Complete Surface*, *Vestibular Ridge* and *Border* no significant differences have been found for precision between the digitalization methods.

### Accuracy of digitalization area:

For PRI and TR4 the significantly highest trueness was exhibited at *Alveolar Ridge*. For TR3 *Complete Surface*, *Alveolar Ridge* and *Vestibular Ridge* showed significantly higher trueness than *Palate, Posterior Seal* and *Border*. For D8I the significantly highest trueness was observed at *Complete Surface and Alveolar Ridge* while *Border* showed the significantly lowest trueness.

For PRI, TR4, TR3 and D8I the significantly highest precision was found in *Complete Surface* and *Alveolar Ridge.*

## Discussion

In the present in vitro study, the accuracy of different digitalization processes as well as the accuracy of digitalization for distinctive areas of the edentulous maxilla were investigated. The first null hypothesis that stated no differences between the digitalization methods could be detected must be rejected. The superior accuracy demonstrated by indirect digitalization of impressions compared to direct digitalization has been confirmed by several authors for dentate as well as for edentulous anatomies [23, 31–33]. Laboratory scanners are less error-prone than IOS since they operate in controlled environments with standardized procedures. The larger optical sensors can capture broader segments with more features, requiring fewer images and reducing stitching inaccuracies [34, 35]. In accordance with current results, recent in vivo investigations presented lower trueness for the direct digitalization with Trios 3 compared to digitalization of PVS impressions [12, 36]. By contrast, Zarone et al. reported a higher trueness and precision for Trios 3 than for conventional impressions, using polysulfide material [24]. Several authors have disclosed similar performance for Trios 3 for impressions and casts in vivo, however these comparisons have not been based on a reference dataset [13, 25].

Disparities in IOS performance have been attributed to multiple factors including optical scanning technology, specific hardware components of each system such as head size, lens, area of the scanning window as well as the software [29, 37, 38]. A larger scanning window facilitates the capture of fewer images that contain more morphological information and can be more efficiently stitched together resulting in improved accuracy [37]. Consequently, the superior performance of TR4 compared to PRI may be attributed to the larger scanning window of the device (19.20 × 16.30 mm compared to 18.0 × 16.10 mm). In a similar setup Osman et al. determined the size of scanning window to affect the precision of the generated dataset [29]. Apart from hardware differences, the older algorithm utilized for image superimposition and processing may account for the poorer accuracy observed for TR3. This is in accordance with the conclusions of Schmalz et al. who found higher deviations for the digitalization with Trios 3 compared to Trios 4 [39]. Conversely, a previous investigation conducted on an edentulous cadaver maxilla found no differences in trueness and precision between Cerec Primescan, Trios 4 and Trios 3 when the same software version has been installed [40].

The second null hypothesis predicting no significant differences between the different areas of the maxillary model must be also rejected. IOS demonstrated higher trueness in *Alveolar Ridge*, which may be attributed to the scan strategy initiating at the maxillary ridge. Similarly, Zarone et al. concluded for edentulous anatomy that an early recording of areas which negatively influence the stitching process can reduce the scanning accuracy [41]. Furthermore, the distinctive morphological features of the edentulous ridge facilitated a more precise image alignment by the algorithm compared to other anatomical regions [42]. By contrast, Gutmacher et al. attributed the superior trueness of IOS digitalization for the palate in comparison to the ridge to the lower resilience of the tissue, presence of rugae, and smaller area but found no significant differences in precision [40]. The improved accuracy of PRI at the *Vestibular Ridge* can be attributed to the “Smart Sensor” technology. This component employs a rapidly moving lens to acquire a large number of images, varying focus in a small amount of time, increasing the scanning depth range enabling efficient correction of the abrupt depth variations in this area [43].

Irrespective of digitalization method *Border* demonstrated the poorest trueness, possibly due to the steep and narrow geometry of the area, which limits access for optical systems and can result in distortion or voids in the generated dataset. In addition, the *Border* is a smooth and rounded surface and lacks distinctive characteristics that may be used by the superimposition algorithm. In congruence with current results, Al Hamad et al. reported higher deviations for the peripheral areas scanned with Trios 4 [44].

In the current study a “localized best fit” alignment was applied on the surface of defined spherical geometries, while all other areas were excluded for the alignment. This method was chosen to ensure that possible disadvantages of the methodology – such as the distribution of local discrepancies over the entire dataset and the underestimation of true inaccuracies – would be minimized [26, 45]. Furthermore, the absolute value of surface point distances between datasets was used to avoid neutralization of error through averaging of positive and negative values [45, 46]. With the current set up it is possible to quantitatively evaluate and compare trueness and precision for different areas of the edentulous maxilla, which was previously mainly assessed by use of color-coded superimposition graphs [12, 23, 28, 29, 42].

The current results must be assessed considering the study design limitations. Firstly, a maxillary PEEK model was used with different reflective properties than oral mucosa. This may potentially bias the results in favor of optical systems [47]. Additionally, the examination was conducted in vitro. However, in clinical settings, factors like saliva, soft tissue mobility, patient movement, as well as spatial limitations can negatively impact the digitalization accuracy for IOS [48]. Additionally, this study focused exclusively on the accuracy of digitizing the edentulous maxilla. The larger area of attached tissues in the palate provides more information for image superimposition, enhancing the scanning accuracy [31]. Further research in a similar set up should be directed also on the digitalization of edentulous mandibles. Furthermore, the present study investigated only the procedure of data acquisition and not the complete manufacturing procedure.

The accuracy threshold necessary for the fabrication of complete dentures is accepted to be within the range of 300—500 μm and pertains to the compressibility of intraoral mucosa [12, 22, 27, 49]. Deviations between the intaglio surface of final prosthesis and the edentulous anatomy within the range of mucosal thickness can be absorbed by the underlying tissues. Larger discrepancies may result in sore spots, insufficient fit, and instability [14, 21]. Presently all investigated methods exhibited accuracy well below the aforementioned value, in accordance with the findings of contemporary research [12, 13, 21, 22, 27, 41]. Regardless of the method, digitalization of the edentulous maxilla produced higher deviations in the peripheral areas of the anatomy, namely *Border* and *Palatal Seal*. Consequently, for CAD/CAM dentures incongruencies between base and the supporting tissue are expected to be more pronounced in the periphery, where corrections or even a reline may be more frequently necessary. Furthermore, for CAD/CAM workflows that require the superimposition of additional data (e.g. CBCT scans, digital set ups, bite rims) with datasets acquired through direct digitalization, a matching over the edentulous ridge may be more reliable than including areas of the palate [50–52]. Bearing in mind the possible advantages and limitations discussed, direct digitalization remains practical in procuring data for preliminary impressions, digital set up or simulation of prosthetic treatment, and implant planning [52–54]. In addition, digitalization by IOS can be a reliable method for the fabrication of immediate dentures, since peripheral and palatal seal are going to be relined after a period of tissue remodeling [55, 56]. IOS are also indicated when conventional impressions are hindered by factors like a strong gag reflex or limited mouth opening [57, 58]. At the same time, in cases of excessive soft tissue mobility and flabby ridges, the intraoral digitalization without exerting any pressure can be considered as advantageous [13, 59]. Still, indirect digitalization of the impression seems to be the most reliable method for the acquisition of the edentulous anatomy, since it demonstrated the best results and includes information about the peripheral seal.

## Conclusions

Considering the limitations of the current study, the following conclusions can be summarized for the digitalization of edentulous jaws:

- The digitalization of edentulous anatomy depends on the morphology of the scanned area. Areas with limited accessibility and unclear surface structure with sparse features generally show higher deviations in the virtual model dataset.
- The indirect digitalization of the impression seems to be still the most accurate and reliable method.
- Digitalization by IOS can be a reliable method for the fabrication of interims and immediate dentures.

## Data Availability

Data sets generated during the current study are available from the corresponding author on reasonable request.

## References

[1] Schweiger J et al (2018) Systematics and concepts for the digital production of complete dentures: risks and opportunities. Int J Comput Dent 21(1):41–56 29610780

[2] Wulfman C et al (2020) Digital removable complete denture: a narrative review. Fr J Dent Med 10:1–9

[3] Janeva N et al (2018) Advantages of CAD/CAM versus Conventional Complete Dentures - A Review. Op Acc Maced J Med Sci, 6 10.3889/oamjms.2018.308 PMC6108805 30159084

[4] Baba NZ et al (2021) CAD/CAM Complete denture systems and physical properties: a review of the literature. J Prosthodont 30(S2):113–124 32844510 10.1111/jopr.13243

[5] AlHelal A et al (2017) Comparison of retention between maxillary milled and conventional denture bases: A clinical study. J Prosthet Dent 117(2):233–238 27765399 10.1016/j.prosdent.2016.08.007

[6] Ender A, Attin T, Mehl A (2016) In vivo precision of conventional and digital methods of obtaining complete-arch dental impressions. J Prosthet Dent 115(3):313–320 26548890 10.1016/j.prosdent.2015.09.011

[7] Malik J et al (2018) Comparison of accuracy between a conventional and two digital intraoral impression techniques. Int J Prosthodont 31(2):107–113 29518805 10.11607/ijp.5643

[8] Abduo J, Elseyoufi M (2018) Accuracy of intraoral scanners: a Systematic review of influencing factors. Eur J Prosthodont Restor Dent 26(3):101–121 29989757 10.1922/EJPRD_01752Abduo21

[9] Renne W et al (2017) Evaluation of the accuracy of 7 digital scanners: An in vitro analysis based on 3-dimensional comparisons. J Prosthet Dent 118(1):36–42 28024822 10.1016/j.prosdent.2016.09.024

[10] Lee JH (2017) Improved digital impressions of edentulous areas. J Prosthet Dent 117(3):448–449 27881330 10.1016/j.prosdent.2016.08.019

[11] Albanchez-González MI et al (2022) Accuracy of digital dental implants impression taking with intraoral scanners compared with conventional impression techniques: a systematic review of In vitro studies. Int J Environ Res Public Health, 19(4) 10.3390/ijerph19042026 PMC8872312 35206217

[12] Lo Russo L et al (2020) Three-dimensional differences between intraoral scans and conventional impressions of edentulous jaws: A clinical study. J Prosthet Dent 123(2):264–268 31153614 10.1016/j.prosdent.2019.04.004

[13] Chebib N et al (2019) Edentulous jaw impression techniques: An in vivo comparison of trueness. J Prosthet Dent 121(4):623–630 30580982 10.1016/j.prosdent.2018.08.016

[14] Rasaie V, Abduo J, Hashemi S (2021) Accuracy of intraoral scanners for recording the denture bearing areas: a systematic review. J Prosthodont 30(6):520–539 10.1111/jopr.13345 33554361

[15] Jacobson TE, Krol AJ (1983) A contemporary review of the factors involved in complete denture retention, stability, and support. Part I: retention J Prosthet Dent 49(1):5–15 6337253 10.1016/0022-3913(83)90228-7

[16] Jacobson TE, Krol AJ (1983) A contemporary review of the factors involved in complete dentures. Part III: support J Prosthet Dent 49(3):306–313 6341544 10.1016/0022-3913(83)90267-6

[17] Lo Russo L, Salamini A (2018) Single-arch digital removable complete denture: a workflow that starts from the intraoral scan. J Prosthet Dent, 120(1):20–24 29195814 10.1016/j.prosdent.2017.09.004

[18] Kattadiyil MT et al (2014) Intraoral scanning of hard and soft tissues for partial removable dental prosthesis fabrication. J Prosthet Dent 112(3):444–448 24882595 10.1016/j.prosdent.2014.03.022

[19] Bohannan HM (1954) A critical analysis of the mucostatic principle. J Prosthet Dent 4(2):232–241

[20] Page HL (1946) Mucostatics, a Principle - Not a "technique"

[21] Srivastava G et al (2023) Accuracy of intraoral scanner for recording completely edentulous arches-a systematic review. Dent J (Basel), 11(10) 10.3390/dj11100241 PMC10605168 37886926

[22] Jung S et al (2019) Comparison of different impression techniques for edentulous jaws using three-dimensional analysis. J Adv Prosthodont 11(3):179–186 31297177 10.4047/jap.2019.11.3.179 PMC6609757

[23] Hack G et al (2020) Computerized optical impression making of edentulous jaws - An in vivo feasibility study. J Prosthodont Res 10.1016/j.jpor.2019.12.003 32061572

[24] Zarone F et al (2020) Accuracy of a chairside intraoral scanner compared with a laboratory scanner for the completely edentulous maxilla: An in vitro 3-dimensional comparative analysis. J Prosthet Dent 124(6):761.e1-761.e7 33289647 10.1016/j.prosdent.2020.07.018

[25] Willmann C et al (2024) Intraoral optical impression versus conventional impression for fully edentulous maxilla: an in vivo comparative study. Int J Comput Dent 27(1):19–26 36815624 10.3290/j.ijcd.b3916775

[26] O’Toole S et al (2019) Investigation into the accuracy and measurement methods of sequential 3D dental scan alignment. Dent Mater 35(3):495–500 30683418 10.1016/j.dental.2019.01.012

[27] Osnes CA et al (2020) Full arch precision of six intraoral scanners in vitro. J Prosthodont Res 64(1):6–11 31227447 10.1016/j.jpor.2019.05.005

[28] Schimmel M et al (2021) Accuracy of intraoral scanning in completely and partially edentulous maxillary and mandibular jaws: an in vitro analysis. Clin Oral Investig 25(4):1839–1847 32812098 10.1007/s00784-020-03486-z PMC7966190

[29] Osman RB, Alharbi NM (2023) Influence of scan technology on the accuracy and speed of intraoral scanning systems for the edentulous maxilla: An in vitro study. J Prosthodont 32(9):821–828 36571837 10.1111/jopr.13633

[30] ISO 5725–1:2023 Accuracy (trueness and precision) of measurement methods and results, in Part 1: General principles and definitions. 2023, International Organization for Standardization

[31] Kuhr F et al (2016) A new method for assessing the accuracy of full arch impressions in patients. J Dent 55:68–74 27717754 10.1016/j.jdent.2016.10.002

[32] Keul C et al (2020) Accuracy of data obtained from impression scans and cast scans using different impression materials. Int J Comput Dent 23(2):129–138 32555766

[33] Schmidt A et al (2020) Accuracy of digital and conventional full-arch impressions in patients: an Update. J Clin Med, 9(3) 10.3390/jcm9030688 PMC7141355 32143433

[34] Keul C, Güth JF (2020) Accuracy of full-arch digital impressions: an in vitro and in vivo comparison. Clin Oral Investig 24(2):735–745 31134345 10.1007/s00784-019-02965-2

[35] Nagy Z et al (2020) Comparing the trueness of seven intraoral scanners and a physical impression on dentate human maxilla by a novel method. BMC Oral Health 20(1):97 32264943 10.1186/s12903-020-01090-x PMC7137345

[36] Kalberer N et al (2021) In silico evaluation of the peripheral and inner seals in complete denture master impressions using a custom-developed 3D software. Clin Oral Investig 25(1):125–132 32488486 10.1007/s00784-020-03343-z

[37] Hayama H et al (2018) Trueness and precision of digital impressions obtained using an intraoral scanner with different head size in the partially edentulous mandible. J Prosthodont Res 62(3):347–352 29502933 10.1016/j.jpor.2018.01.003

[38] Kim JE et al (2017) Accuracy of intraoral digital impressions using an artificial landmark. J Prosthet Dent 117(6):755–761 27863856 10.1016/j.prosdent.2016.09.016

[39] Schmalzl J et al (2023) The effect of generation change on the accuracy of full arch digital impressions. BMC Oral Health 23(1):766 37853398 10.1186/s12903-023-03476-z PMC10585882

[40] Gutmacher Z et al (2021) Evaluation of the accuracy of multiple digital impression systems on a fully edentulous maxilla. Quintessence Int 52(6):488–495 33880909 10.3290/j.qi.b1244373

[41] Zarone F et al (2020) Comparison of different intraoral scanning techniques on the completely edentulous maxilla: an in vitro 3-dimensional comparative analysis. J Prosthet Dent 124(6):762.e1–762.e8 10.1016/j.prosdent.2020.07.017 33289648

[42] Osman R, Alharbi N (2022) Does the palatal vault form have an influence on the scan time and accuracy of intraoral scans of completely edentulous arches? An in-vitro study J Adv Prosthodont 14(5):294–304 36452365 10.4047/jap.2022.14.5.294 PMC9672697

[43] Falih MY, Majeed MA (2023) Trueness and Precision of Eight Intraoral Scanners with Different Finishing Line Designs: A Comparative In Vitro Study. Eur J Dent 17(4):1056–1064 36513335 10.1055/s-0042-1757568 PMC10756783

[44] Al Hamad KQ, Al-Kaff FT (2023) Trueness of intraoral scanning of edentulous arches: a comparative clinical study. J Prosthodont, 32(1)26–31 35997079 10.1111/jopr.13597

[45] Güth JF et al (2016) A new method for the evaluation of the accuracy of full-arch digital impressions in vitro. Clin Oral Investig 20(7):1487–1494 26454734 10.1007/s00784-015-1626-x

[46] D'Arienzo LF, D€™Arienzo A, Borracchini A (2018) Comparison of the suitability of intra-oral scanning with conventional impression of edentulous maxilla in vivo. A preliminary study. J Osseointegration, 10(4):115–120

[47] van der Meer WJ et al (2012) Application of intra-oral dental scanners in the digital workflow of implantology. PLoS ONE 7(8):e43312 22937030 10.1371/journal.pone.0043312 PMC3425565

[48] Flügge TV et al (2013) Precision of intraoral digital dental impressions with iTero and extraoral digitization with the iTero and a model scanner. Am J Orthod Dentofacial Orthop 144(3):471–478 23992820 10.1016/j.ajodo.2013.04.017

[49] Picton DC, Wills DJ (1978) Viscoelastic properties of the periodontal ligament and mucous membrane. J Prosthet Dent 40(3):263–272 100600 10.1016/0022-3913(78)90031-8

[50] Carosi P et al (2020) Digital workflow to merge an intraoral scan and CBCT of edentulous Maxilla: a technical report. J Prosthodontics, 29 10.1111/jopr.13221 32608078

[51] Gallucci GO et al (2015) Innovative approach to computer-guided surgery and fixed provisionalization assisted by screw-retained transitional implants. Int J Oral Maxillofac Implants 30(2):403–410 25830401 10.11607/jomi.3817

[52] Meneghetti P et al (2021) A fully digital approach for implant fixed complete dentures: A case report. J Esthet Restor Dent 33(8):1070–1076 34213055 10.1111/jerd.12798

[53] Elnagar MH, Aronovich S, Kusnoto B (2020) Digital workflow for combined orthodontics and orthognathic surgery. Oral Maxillofac Surg Clin North Am 32(1):1–14 31699582 10.1016/j.coms.2019.08.004

[54] Flügge T et al (2022) Digital implantology-a review of virtual planning software for guided implant surgery. Part II: Prosthetic set-up and virtual implant planning. BMC Oral Health, 22(1):23 35094677 10.1186/s12903-022-02057-w PMC8802526

[55] Mendonça G et al (2021) Digital immediate complete denture for a patient with Rhabdomyosarcoma: a clinical report. J Prosthodont 30(3):196–201 33325048 10.1111/jopr.13305

[56] Fang JH et al (2018) Digital immediate denture: A clinical report. J Prosthet Dent 119(5):698–701 28927924 10.1016/j.prosdent.2017.06.004

[57] Wu J, Li Y, Zhang Y (2017) Use of intraoral scanning and 3-dimensional printing in the fabrication of a removable partial denture for a patient with limited mouth opening. J Am Dent Assoc 148(5):338–341 28274480 10.1016/j.adaj.2017.01.022

[58] Londono J et al (2015) Fabrication of a definitive obturator from a 3D cast with a chairside digital scanner for a patient with severe gag reflex: a clinical report. J Prosthet Dent 114(5):735–738 26182852 10.1016/j.prosdent.2015.01.019

[59] Tripathi A et al (2019) Effect of mucostatic and selective pressure impression techniques on residual ridge resorption in individuals with different bone mineral densities: A prospective clinical pilot study. J Prosthet Dent 121(1):90–94 30006216 10.1016/j.prosdent.2018.02.016

